# Diversifying Implementation Science: A Global Perspective

**DOI:** 10.9745/GHSP-D-21-00757

**Published:** 2022-08-30

**Authors:** Sophia M. Bartels, Shabab Haider, Caitlin R. Williams, Yameen Mazumder, Latifat Ibisomi, Olakunle Alonge, Sally Theobald, Till Bärnighausen, Juanita Vasquez Escallon, Mahnaz Vahedi, Rohit Ramaswamy, Malabika Sarker

**Affiliations:** a Department of Health Behavior, The University of North Carolina Gillings School of Global Public Health, Chapel Hill, NC, USA.; b James P Grant School of Public Health, BRAC University, Dhaka, Bangladesh.; c Department of Maternal and Child Health, The University of North Carolina Gillings School of Global Public Health, Chapel Hill, NC, USA.; d Division of Epidemiology and Biostatistics, School of Public Health, University of the Witwatersrand, Johannesburg, South Africa.; e Nigerian Institute of Medical Research, Lagos, Nigeria.; f Department of International Health, Johns Hopkins University Bloomberg School of Public Health, Baltimore, MD, USA.; g Social Science and International Health, Liverpool School of Tropical Medicine, Liverpool, United Kingdom.; h Heidelberg Institute of Global Health, Faculty of Medicine and University Hospital, Heidelberg, Germany.; i UNICEF, New York, NY, USA.; j World Health Organization, Geneva, Switzerland.; k James M. Anderson Center for Health Systems Excellence, Cincinnati Children's Hospital Medical Center, Cincinnati, OH, USA.

## Abstract

We present a joint global perspective about the urgent need to diversify the loci of knowledge creation and sharing in global implementation science. We underscore the imperative of addressing implementation research questions relevant to practitioners, policy makers, and researchers from low- and middle-income countries.

## Key Messages

- As part of the greater movement to decolonize global health, leadership in implementation science needs to diversify to increase the influence of researchers and practitioners from low- and middle-income countries (LMICs) and to accelerate the development of implementation research suited to LMICs.
- Addressing this need will require the engagement of policy makers in LMICs to fund and shape the local research agenda, the creation of global networks to adapt or develop implementation science frameworks aligned with LMIC settings, the advancement of peer-to-peer learning and LMIC-based mentorship, and the prioritization of equity in implementation research.
- Achieving these aspirations will require cooperation across regions and continents to diversify global leadership in the field.

## INTRODUCTION: ALIGNING IMPLEMENTATION SCIENCE WITH GLOBAL HEALTH DECOLONIZATION

Implementation science, the “scientific study of methods and strategies that facilitate the uptake of evidence-based practice and research into regular use by practitioners and policy makers,”^1^ has been recognized as a key approach to accelerate progress toward achieving the Sustainable Development Goals (SDGs).^2^ In the past few years, there has been significant interest in the use of implementation science to advance the effectiveness and sustainment of global health programs.^3^ The field has roots in diffusion of innovation, knowledge utilization, and technology transfer and is influenced by other traditions, such as health behavior, political science, organizational behavior, and systems science.^4^^–^^6^ Foundational research in all of these fields has been primarily led by researchers at academic institutions in high-income countries (HICs), and the principles and findings have spread to global settings.^4^ As a result, the leading theories, models, and frameworks (TMF) in implementation science have also been primarily developed in HICs, have been globally exported, and are the guiding principles for implementation research and practice worldwide. Training programs around the world present these principles as a single unified body of knowledge despite the acknowledgment that research on implementation explicitly needs to account for heterogeneity in contexts, systems, and settings, and that implementation science concepts and principles need to be adapted or re-invented for low- and middle-income country (LMIC) settings.^2^^,^^7^^–^^10^

Recently, HIC dominance in global health research and practice has again been critiqued under the banner of “decolonizing global health.”^11^^–^^13^ In reflecting on the lessons of 2020, the United Nations Special Rapporteur on the Right to Health, Dr. Tlaleng Mofokeng, issued a call to “end the colonialism and racism embedded in the global health architecture.”^14^ While debate remains about the definition, scope, and aims of “decolonization” in the context of global health, the literature on this topic critiques how the practice of global health has reinforced colonial patterns.^15^ Such patterns are evidenced through HIC dominance in defining research priorities, controlling funding, restricting access to knowledge (e.g., through paywalls and editorial and financial structures that favor publications in a few HIC-based high-impact journals), and establishing 1-way flows of expertise under the label of capacity building.^16^ As decolonial thinkers have long highlighted, HIC epistemologies, though cloaked in “universalism,” have actually served to simultaneously render invisible and advance the primacy of HIC authority and power.^17^ Ensuring equal footing for a plurality of epistemologies and worldviews (Grosfoguel’s “pluri-versalism”)^18^ can serve as one possible counterweight. Authors from LMICs have also pointed out that ironically, the rhetoric on decolonization has also been led by HICs, creating a mistaken impression that practitioners and institutions from LMICs are not seriously engaged in these efforts.^19^

Calls from the global health field for transforming how research agendas are set, funding is distributed, and expertise is created and disseminated—balanced with a pragmatic approach led by those who have the greatest stake in the results of this transformation—are equally germane for implementation science. As the field increases in popularity, its practitioners need to scrutinize the applicability of implementation science research foci, methods, and core competencies developed in HICs to LMIC settings^7^^,^^8^^,^^20^^–^^22^; interrogate the ability of implementation research to address issues of power and inequality in implementation within and across countries^23^^,^^24^; and increase diversity of research, practice, and policy leadership in the field. These considerations will facilitate a vision of implementation science in which the power and resource imbalances described in the decolonization literature that impede shared research, practice, and policy leadership between HICs and LMICs cease to exist. However, the impetus for these efforts must come from LMICs and must not be driven by the prevailing preoccupations of HICs. In this article, we use a global health decolonization lens to frame our call for diversification of research leadership in implementation science so that the knowledge to address critical implementation challenges is generated and applied closer to where implementation actually occurs.

Implementation science practitioners need to interrogate the ability of implementation research to address issues of power and inequality within and across countries.

The Global Conference for Implementation Science (GCIS), held in Dhaka, Bangladesh, June 29–July 1, 2019, was the field’s first global conference organized by an LMIC. The organizers invited individuals from HICs and LMICs involved in implementation science training and research in LMICs to combine their personal experiences with perspectives provided by the conference attendees on how to make the field more relevant to their settings. This commentary is the result of that invitation. All the authors are affiliated with academic institutions or with international donor organizations. We have been deeply involved in funding educational programs, developing curricula, or teaching and mentoring students, researchers, and practitioners from LMICs in implementation science and have been personally challenged in helping students apply the theories and frameworks we teach to their settings. The themes surfaced by the conference attendees align with our experience and reinforce our call to action.

## IMPLEMENTATION SCIENCE FOR LMICS: KEY THEMES FROM CONFERENCE ATTENDEES

The GCIS conference was held at the Centre of Excellence for Science of Implementation and Scale-Up at the BRAC James P Grant School of Public Health to create a space for researchers, practitioners, students, and policy makers to share learning, discuss the value of implementation science and research in LMIC settings, and provide recommendations on how the field should serve the research needs of LMICs. It was cosponsored by BRAC University, UNICEF Bangladesh, and the World Health Organization (WHO)’s Special Programme for Research and Training in Tropical Diseases (TDR). More than 250 delegates from 30 countries attended, with 80% of participants from LMICs.^25^ The conference also served as the venue for the annual meeting of faculty from 7 universities in Asia, Africa, Latin America, and the Middle East that had received WHO funds to establish graduate programs in implementation science.

To build on the momentum of the conference, the conference organizers at BRAC University sent conference attendees an online survey to solicit feedback on the conference and generate ideas for the future. For the latter aim, respondents were asked to list 1 priority action needed to promote LMIC leadership in implementation science and 1 priority action to develop implementation research methods relevant to LMIC settings. The 81 responses were tabulated and analyzed by a graduate student at BRAC University in Bangladesh. A University of North Carolina graduate student then used thematic analysis to aggregate the data into coherent themes following the recommendations by Braun et al. that researchers should use thematic analysis in a “knowing way” to “produce an overall coherent piece of work.”^26^ We present these themes framed through the lens of global health decolonization.

### Theme 1: Policy Makers in LMICs Do Not Set Research Agendas or Fund Implementation Research

Conference participants emphasized the need for engagement strategies to motivate policy makers to adopt and fund implementation research agendas in their respective countries. These strategies should encourage the involvement of policy makers in setting implementation research priorities, providing funding for researcher-practitioner collaborations to conduct research based on these priorities, and ensuring the dissemination and application of these findings in policy and practice.^27^ Lack of involvement of local policy makers may perpetuate the power asymmetries that have traditionally characterized global partnerships by allowing international researchers rather than local policy makers to set research priorities.^28^^,^^29^

Lack of involvement of local policy makers may perpetuate the power asymmetries that have traditionally characterized global partnerships by allowing international researchers rather than local policy makers to set research priorities.

In addition, participants expressed a desire for more opportunities to train policy makers in their settings to help them understand how implementation research can provide practitioners with actionable strategies for effective dissemination and scale-up. The implementation science literature has articulated the importance of the “virtuous cycle”^30^ in which research informs better practice, and nongovernmental organizations and other implementers partner with researchers to set local research priorities to determine the best strategies for capacity building and implementation support. Helping policy makers understand this loop will assist in creating a rationale for them to engage in supporting and sponsoring necessary research on country-specific implementation challenges. Without this training, decisions to scale up tend to be made without the requisite planning for capacity building or implementation support, resulting in disparate outcomes and poor sustainment.

### Theme 2: Implementation Science TMFs Are Primarily Developed in HICs

Participants perceived implementation science TMFs in the current literature as excessively complex and difficult to apply to their situations. Participants felt that the exportation of frameworks developed in HICs to LMICs was conducted with limited research about their utility and need for adaptation and that this led to challenges for LMIC practitioners as they attempted to apply them on the ground. They reinforced the need for greater leadership from LMIC researchers and practitioners to assess when HIC-developed methodologies and theories are relevant, when they need to be tested and adapted, and when local methods need to be developed. They felt that this research has relevance beyond LMICs because resource constraints may drive the creation of novel implementation approaches leading to “reverse innovations”^31^ with universal utility. Since the conference, while studies have been published on the adaptation of the Consolidated Framework for Implementation Research (CFIR) and Reach, Effectiveness, Adoption, Implementation, Maintenance (RE-AIM) to low-resource settings, these have been led by researchers from HIC institutions.^8^^,^^32^

### Theme 3: HICs Monopolize Opportunities for Knowledge Sharing

GCIS participants expressed concerns that while groundbreaking research in implementation science occurs in LMICs, opportunities to showcase more locally relevant knowledge from these settings are limited. Almost all the well-known implementation science conferences take place in HICs, and the editorial boards of existing implementation research-focused journals are heavily represented by HICs. Many of the participants had been trained in content developed in HICs and delivered by instructors from these settings. They welcomed the opportunity that the GCIS conference provided to meet other researchers from LMICs; to discover how they were using implementation science approaches, tools, and methods in their settings and research areas, as well as what aspects of their training worked best in LMIC settings; and to learn how to network and collaborate in environments where local funding for implementation research is often unavailable.

While groundbreaking research in implementation science occurs in LMICs, opportunities to showcase more locally relevant knowledge from these settings are limited.

### Theme 4: Equity and Community Participation Are Not Currently Emphasized in the Implementation Research Agenda

GCIS participants named equity and methods for including community members who are the recipients of programs or interventions as priority topics for furthering implementation research in LMIC contexts. This was also highlighted in the recently developed core competencies framework for implementation research in LMICs,^7^ where the authors noted that while the HIC competency frameworks emphasize “IR [Implementation Research] theories, methods and designs,” core competencies in LMICs should also include knowledge about “contexts, health systems, ethics, equity issues, communication and advocacy skills.”^7^ Despite some progress in equity-focused frameworks, mainstream implementation research has been largely equity-agnostic, with an assumption that improving the quality and fidelity of implementation will also rectify inequities.^33^^,^^34^

## CREATING A DIVERSE AND GLOBAL IMPLEMENTATION SCIENCE OF THE FUTURE

Input from conference participants reinforces our belief that urgent action is needed to create a decolonizing path for the future of implementation science. To envision the potential future that might result from this effort, we turn to an article by Abimbola and Pai in which they imagine the future “promised land” of academic global health in which supremacy in global health practice has disappeared.^16^ We use selected quotes from this article to articulate our own aspirations for a transformation in implementation science.

We believe urgent action is needed to create a decolonizing path for the future of implementation science.

### Imagining the Future 1: Decentralized Knowledge Creation

*In this imagined future, global health practitioners in HICs and those who are otherwise privileged, have embraced an appropriately modest view of their importance and mastered the art of critical allyship, where they see their primary role as allies and enablers rather than leaders.* ^16^

A global field of implementation science will shift influence from academic researchers in HICs to a diverse coalition of researchers and practitioners worldwide. This will happen through 2-way partnerships between LMICs and HICs in the form of implementation science networks involving academia, government agencies, and nongovernmental implementing partners. The role of these networks will be to jointly develop the capability of LMIC partners to spearhead the creation of context-appropriate theories and methods, with researchers from HICs actively promoting the emergence of leadership from LMICs, ultimately learning from them in the future. An example of such a network is the partnership between Wits University in South Africa, the University of North Carolina at Chapel Hill, USA, and various South African implementation partners.^35^ This network began with the development of a graduate program in implementation science at Wits University but has since provided learning opportunities for University of North Carolina students under the mentorship of South African partners.

### Imagining the Future 2: Equitable Access and Opportunity for Learning and Knowledge Sharing

*Imbalance in authorship within partnerships between HICs and LMICs is a thing of the past. Journals have been transformed. Knowledge platforms are now decentralised and democratised. No longer exclusive, high-impact western journals now exist among a multitude of go-to places, most of which are now based in the Global South. In our reimagined world, the traditional mindset in global health—that expertise flows from HICs to LMICs—is a thing of the past.* ^16^

In this imagined future, practitioners and researchers in HICs and LMICs will form communities of practice or peer-to-peer collaborative learning networks.^36^^,^^37^ Such networks will support global knowledge creation in the form of tools and strategies relevant to a variety of global contexts, provide frictionless opportunities to share knowledge, and facilitate the diffusion of knowledge among researchers in LMICs and between researchers in LMICs and HICs. They will be actively engaged in creating funding opportunities for LMIC-led conferences such as the GCIS and the recent conference on Implementation Science for Cancer Control in Africa.^38^ An equal partnership between HICs and LMICs in mentoring and sharing will benefit both groups. Researchers from HICs can learn from those with deep contextual knowledge and lived experience, while those from LMICs can enhance their technical expertise. They can also use their influence to tackle challenges in knowledge sharing arising from factors such as barriers in researcher-practitioner partnership, the dominance of English as the language of dissemination, or journal word limits that inhibit the detailed description of implementation processes. An early example of this type of partnership is the Fogarty International Center-funded Adolescent HIV Implementation Science Alliance, which was created in 2017 to facilitate knowledge sharing between the National Institute of Health-funded research and local implementing partners.^39^

### Imagining the Future 3: Research Driven by Local Policy Makers Trained in Implementation Science

*These organisations are no longer White-led, White-dominated institutions in HICs but have reoriented their operations to be closer and accountable to the people they serve. They are run by people who are local to the issues and local knowledge takes pre-eminence.* ^16^

In a diversified field of implementation science, funding from HICs will prioritize training and capacity building for policy makers that will enable their close connection to the implementation process and their understanding of the value and need for implementation research. As a result, policy makers will commit to ensuring that implementation research drives policy decisions and facilitates the expansion of the financial base of local institutions to fund implementation research. An example of how this might work is an obstetric triage program that is ready for national scale-up in Ghana. Following the intervention development and an implementation pilot, an externally funded project expanded the program to 8 additional facilities using a systematic, theory-driven implementation science approach.^40^^,^^41^ A technical advisory group consisting of policy makers from the Ghana Health Service partnered with the implementing organization and were trained in the implementation model. Members of the group have now taken ownership of national scale-up using knowledge gained from the implementation research.

In a diversified field of implementation science, funding from HICs will prioritize training and capacity building for policy makers that will enable their close connection to the implementation process and their understanding of the value and need for implementation research.

Another example is a scale-up of kangaroo mother care in Ethiopia and India, where a local research organization partnered with state and local governments to adapt the kangaroo mother care intervention using a customized implementation framework.^10^ Over time, the kind of reverse innovation that has been envisioned will begin to take place, where policy makers from LMICs will be able to influence the global research agenda and funding streams by training researchers from both HICs and LMICs to focus on the most pressing priorities in their settings.

### Imagining the Future 4: Implementation Research Centered Around the Voices of the Vulnerable

*In this future that we can barely see, diversity and inclusiveness are not enough. In this imagined world, representation is as important as how it alters the agenda; what is on the table is as important as who is around the table. It is a landscape that serves the most disadvantaged and recognises that you cannot truly help or support people, be their allies and enablers, without seeing the world through their eyes and seeing yourself as they see you.* ^16^

The past few years have shown an increased recognition of the need for integrating equity into implementation science through the creation of new frameworks with a focus on equity, but these efforts have been led by researchers based in HIC settings.^42^^,^^43^ Recent efforts have also been made to integrate human-centered design ideas into implementation science^44^ to facilitate greater inclusion of stakeholder voices in this field. In the future, implementation research centered on equity will include marginalized communities in HICs and LMICs as codesigners and co-implementers in producing change and will explicitly incorporate the knowledge of those with lived experience of the complex root causes of inequities in settings around the world.^33^ Research will focus on implementation strategies to address structural factors that result in inequitable uptake of programs and interventions, and on the implementation of interventions intended to reduce inequity. Mainstream implementation science will incorporate participatory, decolonial, and liberatory research methods developed by those in LMICs to encourage a diversification of the voices that contribute to implementation science knowledge.^45^^–^^47^

## CONCLUSION

As the first LMIC-organized global conference on implementation science, the GCIS provided an invaluable forum for researchers and practitioners to share perspectives on how to make the field relevant to their contexts, and, by extension, envision a future for the field that benefits everyone. Realizing this future will require a truly global approach, as the 1-way flow of expertise and leadership is replaced by networks of decentralized research leadership. International organizations, such as the WHO and UNICEF, have an important role to play in facilitating regional and global cooperation, advocating for implementation research to meet SDG goals, and providing resources for implementation science capacity building, workshops, and seminars. These organizations have already on this challenge.

The GCIS was sponsored by the WHO’s TDR and UNICEF. TDR has created an implementation research training program across LMICs through partnerships with 7 universities focusing on training and providing opportunities for networking, sharing resources, and providing mentorship to early-career implementation researchers from LMICs.^20^ But, in a field where “context matters”^48^ and local knowledge and expertise play a crucial role, relying on external players is not enough. Policy makers, researchers, and practitioners from LMICs and HICs must make implementation science a local and global priority. In the 2021 SDG achievement report, not a single sub-Saharan African country had achieved more than 65% of the goals.^49^ If we are to realize the SDG vision by 2030, implementation science must evolve from a discipline practiced and promoted by a small number of HIC researchers to becoming part of everyday practice around the world. We need to move a field designed to address the know-do gap from the ivory tower of academic research to the places where knowledge needs to be created and utilized. The time for action is now.

## Notes

**Peer Reviewed**

**First published online:** July 22, 2022.
